# Pain Relief in Patients Undergoing Tonsillectomy

**DOI:** 10.5812/atr.10224

**Published:** 2013-06-01

**Authors:** Francesca G Iodice, Giuseppina Testa

**Affiliations:** 1 Children's Hospital Bambino Gesù, Rome, Italy

**Keywords:** Diclofenac, Gabapentin, Tonsillectomy

*Dear Editor,*

It is with great interest that I read the research article by Dr. Mogadam and colleagues: Comparison of Analgesic Effect between Gabapentin and Diclofenac on Post-Operative Pain in Patients Undergoing Tonsillectomy (1). This randomized controlled trial of 90 patients undergoing tonsillectomy attempts to underline the importance of preemptive analgesia. This involves the beginning of an analgesic regimen before the onset of a noxious stimulus to prevent central sensitization and limit the subsequent pain experience (2). Preemptive analgesia is a treatment that initiated before a surgical procedure reduces the physiological consequences of nociceptive transmission provoked by the nociceptive stimuli. Owing to this ‘protective’ effect on the nociceptive pathways, preemptive analgesia has the potential to be more effective than a similar analgesic treatment initiated after surgery. Consequently, immediate postoperative pain may be reduced and the development of chronic pain may be prevented (3). Pain relief was administered 1 hour before surgery in both the diclofenac and gabapentin groups. As predicted pain scores were lower among the pain relief groups compared with the study group, the authors may have considered comparing a third analgesic regimen, for example paracetamol, rather than a control group (without analgesia). This is more appropriate from an ethical point of view. The author’s choice of gabapentin appears to be adequate, since an increasing number of trials have shown its efficacy as a postoperative analgesic (4). Jeon et al. published an interesting study evaluating the effectiveness of premedication using gabapentin on postoperative pain control in patients undergoing tonsillectomy (5). This randomized trial was performed in adults who were randomly assigned to a control, placebo group and a gabapentin group. Pain assessment was performed using a visual analog scale during resting periods (rVAS) and during swallowing (sVAS) for a period of 9 days after the operation. The number of diclofenac sodium injections and the total amount of fentanyl injected decreased significantly in the gabapentin group (P < 0.01). The sVAS of the gabapentin group was also significantly lower than that of the control group at 2 and 4 h after surgery, but there were no significant differences in the sVAS observed between the two groups for the remainder of the postoperative period. In this study, premedication with gabapentin decreased post-tonsillectomy pain. The authors used only VAS scores to evaluate pain. It should be emphasized that VAS scores and other measures of pain may be influenced by side effects and other confounding variables, and may not be reliable as the sole measure in the evaluation of efficacy of pain medication. In addition, the use of VAS is an assessment that is highly subjective, these scales are of most value when looking at change within individuals, and are of less value for comparing across a group of individuals at one time point.
